# Supplementary material for: Experimental mining plumes and ocean warming trigger stress in a deep pelagic jellyfish
Source: Nat Commun. 2023 Nov 21;14:7352. doi: 10.1038/s41467-023-43023-6 (PMC10663454; doi:10.1038/s41467-023-43023-6)
Supplement: Supplementary file 1 — Supplementary Information [file 41467_2023_43023_MOESM1_ESM.pdf]

Supplementary Materials for

Experimental mining plumes and ocean warming trigger stress in a deep pelagic jellyfish

Vanessa I. Stenvers[1,2*†], Helena Hauss[1,3†], Till Bayer[1], Charlotte Havermans[4], Ute Hentschel[1], Lara Schmittmann[1], Andrew K. Sweetman[5], Henk-Jan T. Hoving[1]

[1] GEOMAR, Helmholtz Centre for Ocean Research Kiel, Düsternbrooker Weg 20, 24105 Kiel, Germany

[2] Department of Invertebrate Zoology, National Museum of Natural History, Smithsonian Institution, Washington, DC 20013, U.S.A.

[3]Norwegian Research Centre AS (NORCE), Stavanger, Norway.

[4] HYIG ARJEL, Functional Ecology, Alfred Wegner Institute Helmholtz Centre for Polar and Marine Research, Am Handelshafen 12, 27570 Bremerhaven, Germany.

[5] Seafloor Ecology and Biogeochemistry Research Group, Scottish Association for Marine Science (SAMS), Oban, Scotland, UK.

[*] Corresponding author: Vanessa I. Stenvers (vstenvers@geomar.de)

[†] These authors contributed equally

Supplementary Methods

Electron Transfer System analysis

ETS activity was analyzed with the Iodonitrotetrazolium reduction assay according to Bode *et al.* [1], based on protocols from Packard[2] and Owens and King[3], with the following modifications. The concentration of polyvinylpyrrolidone (PVP) in the homogenization buffer comprised 0.5 mg mL^{-1}, and the 2-(4-Iodophenyl)-3-(4-nitrophenyl)-5-phenyl-2H-tetrazolium chloride (INT) solution comprised 2.5 mM with pH 7.7. The substrate buffer contained 1.3 mM NADH, 0.05 mM NADPH and 1.0 mM sodium succinate (pH 8.1). For each sample, ~200 to 500 g of frozen bell tissue was subsampled (avoiding internal organs) into pre-weighed Eppendorf tubes and kept on ice to semi-thaw. Ice-cold homogenization buffer was added to each sample in amounts equal to three times the sample volume (e.g. 200 mg tissue in 600 µL buffer)[4]. A 100 mg mixture of glass beads (0.5 and 1.0 mm in diameter) was added to each sample for three rounds of homogenization (15s) in a cell mill followed by 3 minutes of cooling on ice to prevent heating of the sample. Homogenates were centrifuged for two subsequent runs at 5000 g and 4°C for 5 minutes. The final reaction volume contained 210 µL substrate buffer, 70 µL INT solution and 70 µL sample supernatant. The change in absorption was measured at 490 nm, at room temperature (21°C), in a multi-scan spectrophotometer (Multiskan Spectrum, Thermo Fisher Scientific) in 30 sec intervals for 25 minutes. To calculate ETS activity, slopes were corrected for a sample blank (210 µL phosphate buffer, 70 µL INT, 70 µL supernatant) and substrate blank (210 µL substrate buffer, 70 µL INT, 70 µL homogenization buffer) with the following equation [4]:

$$ETS\ (U\ g^{-1}\ WW) = \frac{\Delta A/min}{\varepsilon \cdot d} \cdot \frac{V.cuvette\ (\mu L)}{V.assayed\ (\mu L)} \cdot \frac{V.hombuffer\ (\mu L)}{WW\ (g)}$$

where ΔA/min is the change in corrected absorbance over time, ε is the molar absorptivity of INT-Formazan, *d* is the path length through the spectrophotometer cell, V.cuvette is the final reaction volume (350 µL), V.assayed is the volume of homogenate assayed (70 µL), V.hombuffer is the volume of homogenization buffer used in homogenization (µL), and WW is the wet mass of the sample (g). To obtain the ETS activity in µmol O_2 min^{-1} g WW^{-1}, ETS values were divided by 0.5. The Arrhenius equation was used to correct ETS values for the *in situ* temperatures, based on an activation energy for zooplankton (i.e. 13.2 kcal mol^{-1})[5]. Finally, all values were scaled based on respiration measured directly through fiber optics, using the mean ratio from individuals where

both ETS and fiber optic measurements were taken (i.e. multiplying by 0.0608; data available on PANGAEA repository https://doi.pangaea.de/10.1594/PANGAEA.957367).

Transcriptome analysis

For the transcriptome analysis, a ~0.5x0.5 cm piece of frozen bell tissue was excised from frozen *Periphylla periphylla* and stored in RNAlater at -80°C until further processing. Total RNA was extracted using the Zymo Quick-RNA™ Minprep Plus kit, following the manufacturer's Blood Cells protocol with following modifications. Tissue samples were separated from RNAlater by briefly dipping the tissue on a Kimwipe in close proximity to a Bunsen burner flame, while sterilizing surfaces and forceps in between samples. Samples were loaded into 2 ml Precellys® tubes (CKMix 1.4/2.8 mm), containing pre-cooled 150 µl DNA/RNA Shield™ (2X concentrate) and 150 µl molgrade water, followed by homogenization for 2x10 seconds at 5000 rpm on a Precellys® Evolution Homogenizer. Incubation with 15µl Proteinase K and 30 µl PK Digestion Buffer occurred on a thermal shake incubator at 450 rpm and 30°C for 30 minutes. In the final step of the protocol, 30 µl of molgrade water was added to each spin column to release the final RNA product.

Microbiome analysis

A ~4x4 cm area of epithelial tissue was scraped off individual jellyfish for DNA extractions with the DNeasy Power Soil Kit (Qiagen, Netherlands). Additional reference samples for seawater (12 biological replicates) and sediment samples (3 biological replicates) were extracted with the same kit. Quality and content of DNA extracts were quantified on a NanoDrop®. To test extraction quality, polymerase chain reactions (PCR) were carried out with the universal primers 27F (5'-AGA GTT TGA TCM TGG CTC AG-3') and 1492R (5'-GGT TAC CTT GTT ACG ACT T-3') followed by gel electrophoresis on a 1% agarose gel. For amplicon sequencing, the variable V3 and V4 regions of the 16s rRNA gene were amplified (~300 bp) in a one-step PCR and dual-barcoding approach[6], using the primers 341F (5'-CCT ACG GGA GGC AGC AG-3')[7] and 806R (5'-GGA CTA CHV GGG TWT CTA AT-3')[8]. PCR and gel electrophoresis were performed for verification of amplicon products, followed by normalization and pooling. Products were sequenced on a MiSeq platform (MiSeqGx, Illumina, USA) with v3 chemistry. No mismatches were allowed for demultiplexing sequences.

Fig. S1.

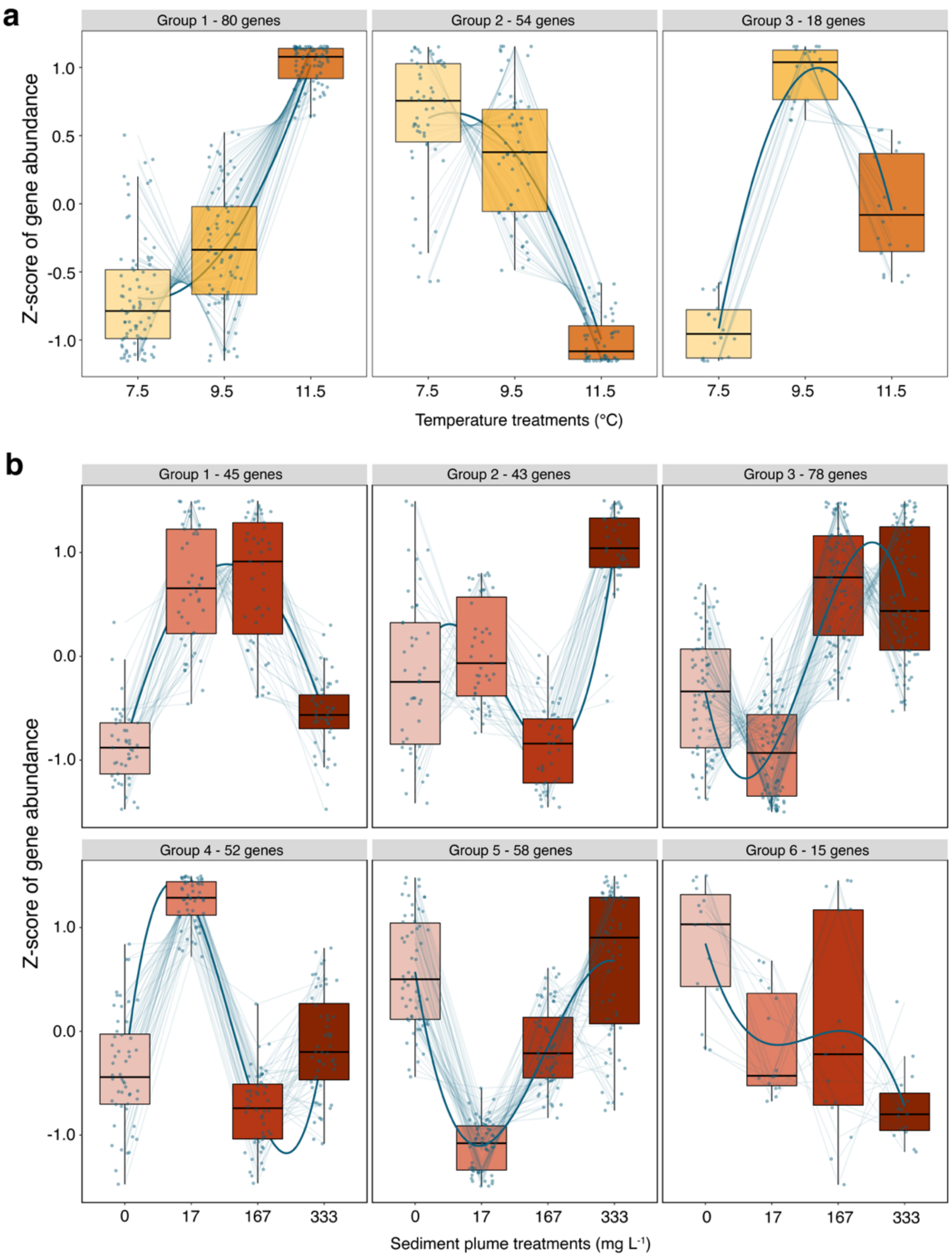

Significantly differentially expressed genes of *Periphylla periphylla* in response to stressor experiments clustered by expression profile. **a** In response to increasing temperatures. **b** In response to increasing sediment concentrations. Profiles were identified using the likelihood ratio test (LRT; adjusted p-value <0.05) in R v4.2.1[9] with the DESeq2 package[10]. Box plots show median (central line) and interquartile (IQR) ranges with whiskers extending to 1.5x the IQR range.

Fig. S2.

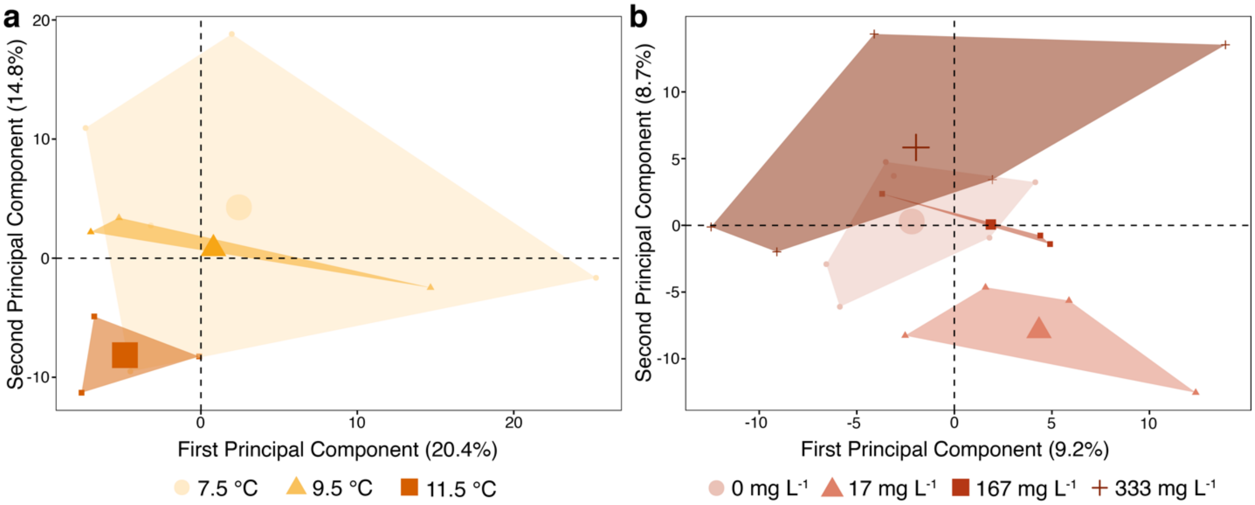

Principal Component Analysis (PCA) of log transformed *Periphylla periphylla* transcriptomes. **a** In response to temperature treatments, including 7.5°C (circles) 9.5°C (triangles) and 11.5°C (squares). **b** In response to suspended sediment, including 0 mg L^{-1} (circles), 17 mg L^{-1} (triangles), 167 mg L^{-1} (squares) and 333 mg L^{-1} (crosses). Mean clustering of treatments is indicated by large shapes in center of each cluster.

Fig. S3.

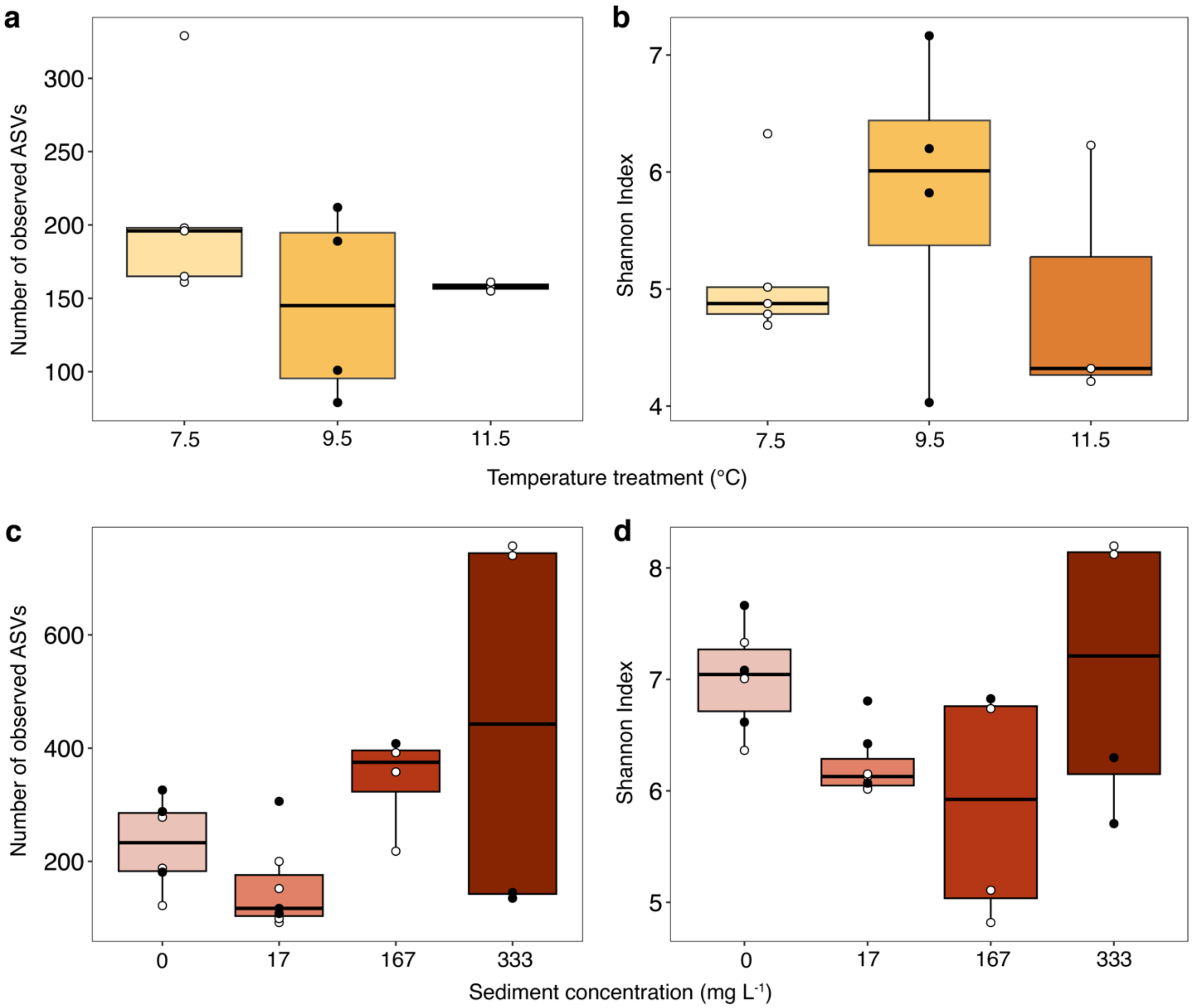

Boxplot of diversity metrics for *Periphylla periphylla* microbiomes exposed to (**a-b**) suspended sediment and (**c-d**) temperature treatments. **a,c** Number of observed amplicon sequence variants (ASVs). **b,d** Shannon Biodiversity index. Data points represent individual specimens, with color indicating sampling date. For **a-b**, these represent 13/03/2021 (white) and 15/03/2021 (black), and for **c-d**, 14/03/2021 (white) and 15/03/2021 (black). Source data are provided in the Source Data file. Box plots show median (central line) and interquartile (IQR) ranges with whiskers extending to 1.5x the IQR range.

Fig. S4.

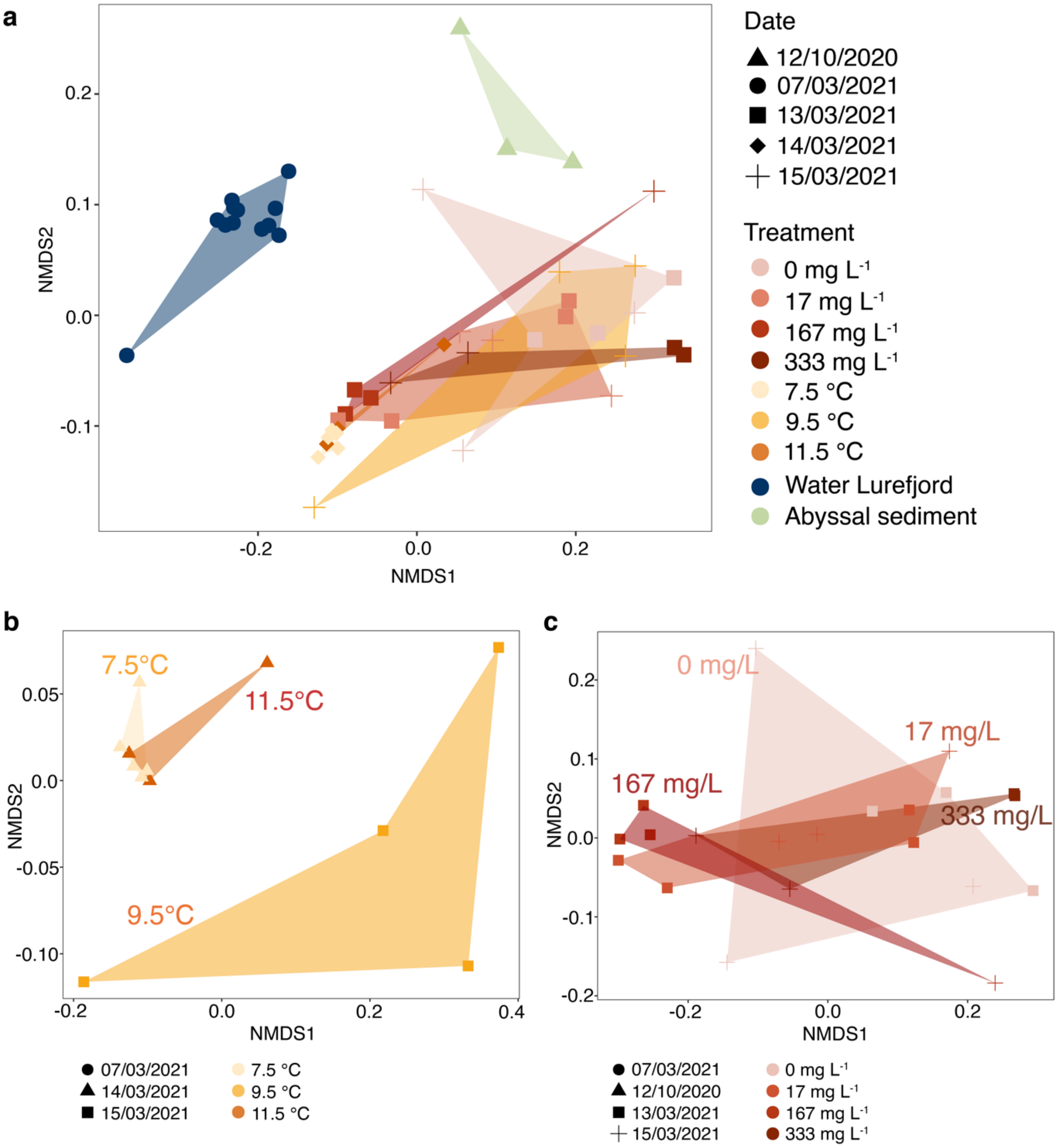

Non-metric multidimensional scaling (NDMS) plots of microbiome composition in *Periphylla periphylla* based on weighed UniFrac distances. **a** Jellyfish microbiomes in response to temperature (yellow colors) and suspended sediment (red colors), compared to Lurefjord seawater (blue) and abyssal plain sediment (green). **b-c** Comparison of microbial community composition within (**b**) temperature and (**c**) sediment treatments. Colors indicate sediment concentration and shapes indicate sampling dates.

Fig. S5.

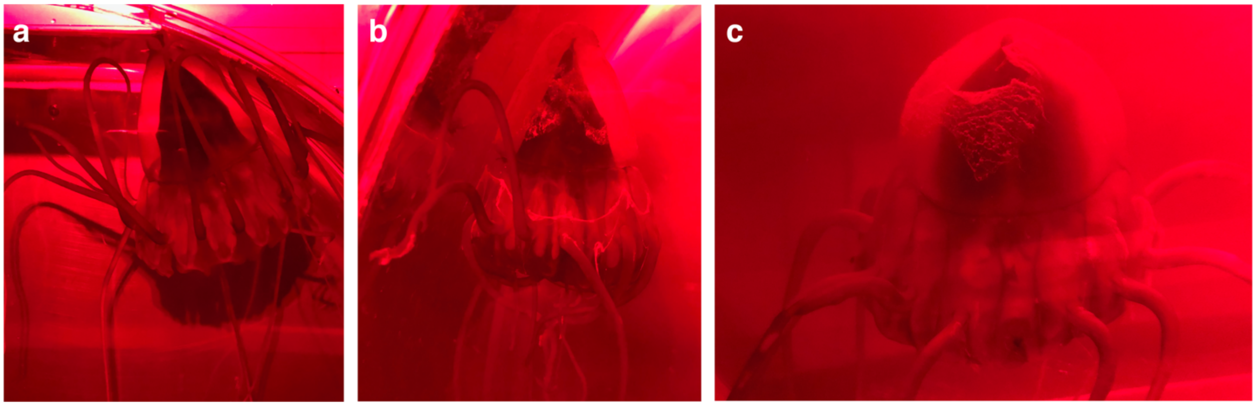

Periphylla periphylla in experimental tanks. **a** Healthy specimen before introduction of suspended sediment (ID 066, 7.31 cm coronal diameter, CD). **b-c** Sediment sloughing off two individuals exposed to 166.7 $mg{\cdot}L^{-1}$. Sediment particles can be seen as white frosting on bell tissue and as white mucous strings on lappets and tentacles. Images taken after ~21 hours (**b**; ID 055, 4.4 cm CD) and 24 hours (**c**; ID 080, 10.2 cm CD) of incubation.

Fig. S6.

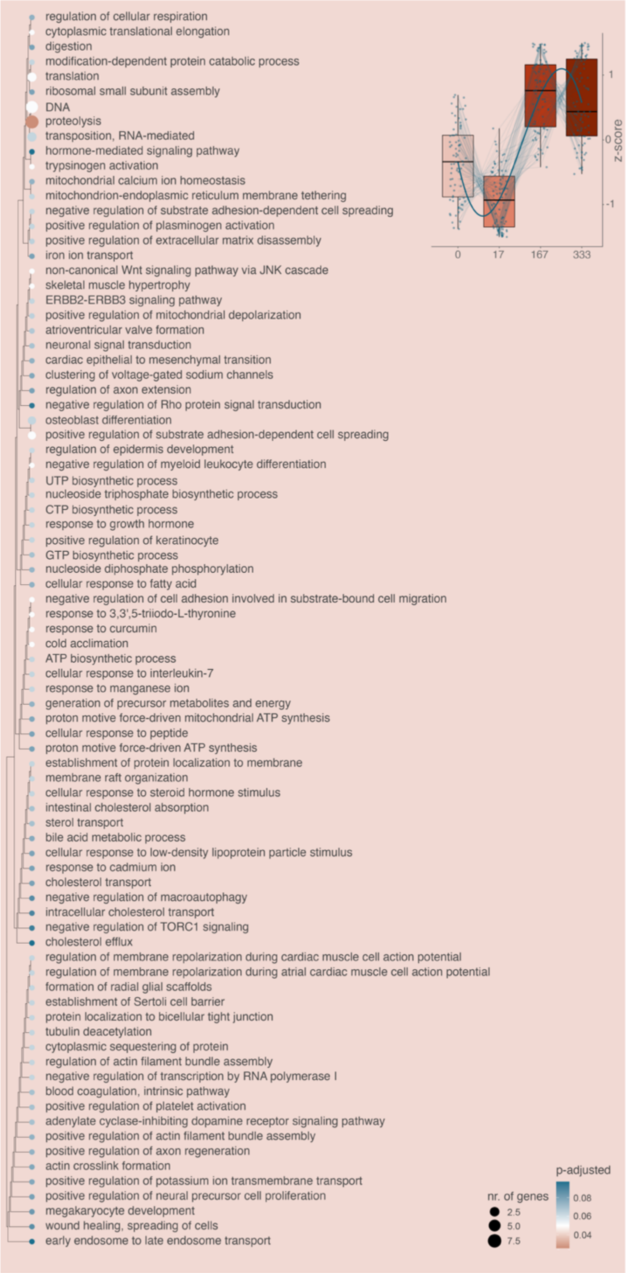

Full gene ontology (GO) of biological processes enriched among significantly overexpressed genes in *Periphylla periphylla* in response to suspended sediment (identified with the Likelihood Ratio Test; n=18 biologically independent samples, from Figure 2d in manuscript). Box plot indicates changes in expression across treatments, taken from Supplementary Fig. S1. Box plot shows median (central line) and interquartile (IQR) ranges with whiskers extending to 1.5x the IQR range. Dot size in the GO tree indicates the number of genes, while colors indicate p-adjusted values (red <0.05, white 0.05, blue > 0.05).

Fig. S7.

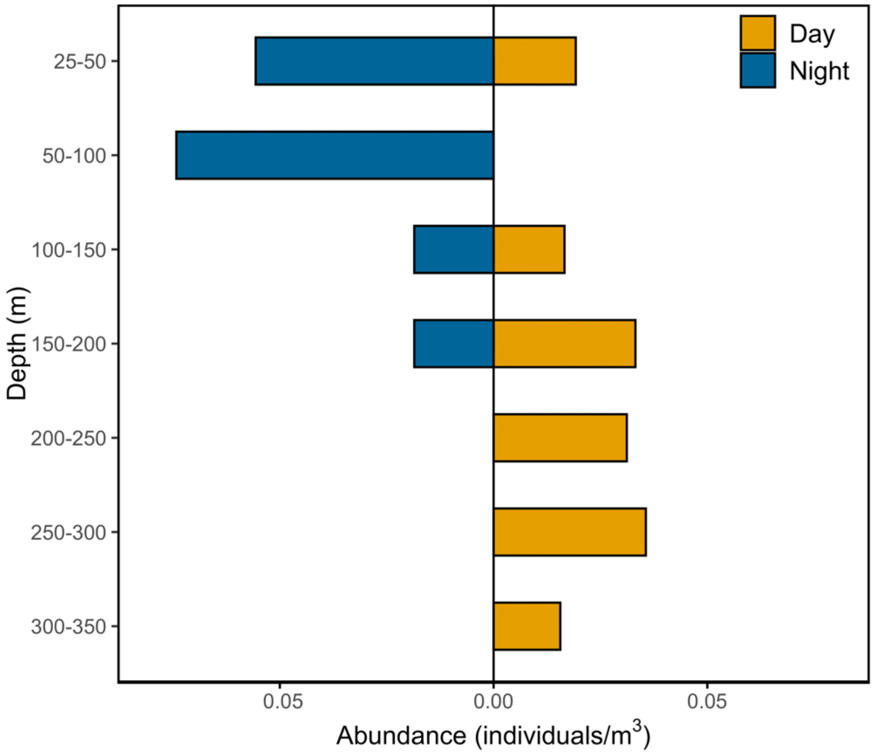

Depth distribution of *Periphylla periphylla* in the Lurefjord, Norway, from multinet catches. Blue indicates night time distribution orange indicates daytime distribution. Source data are provided in the Source Data file.

Fig. S8.

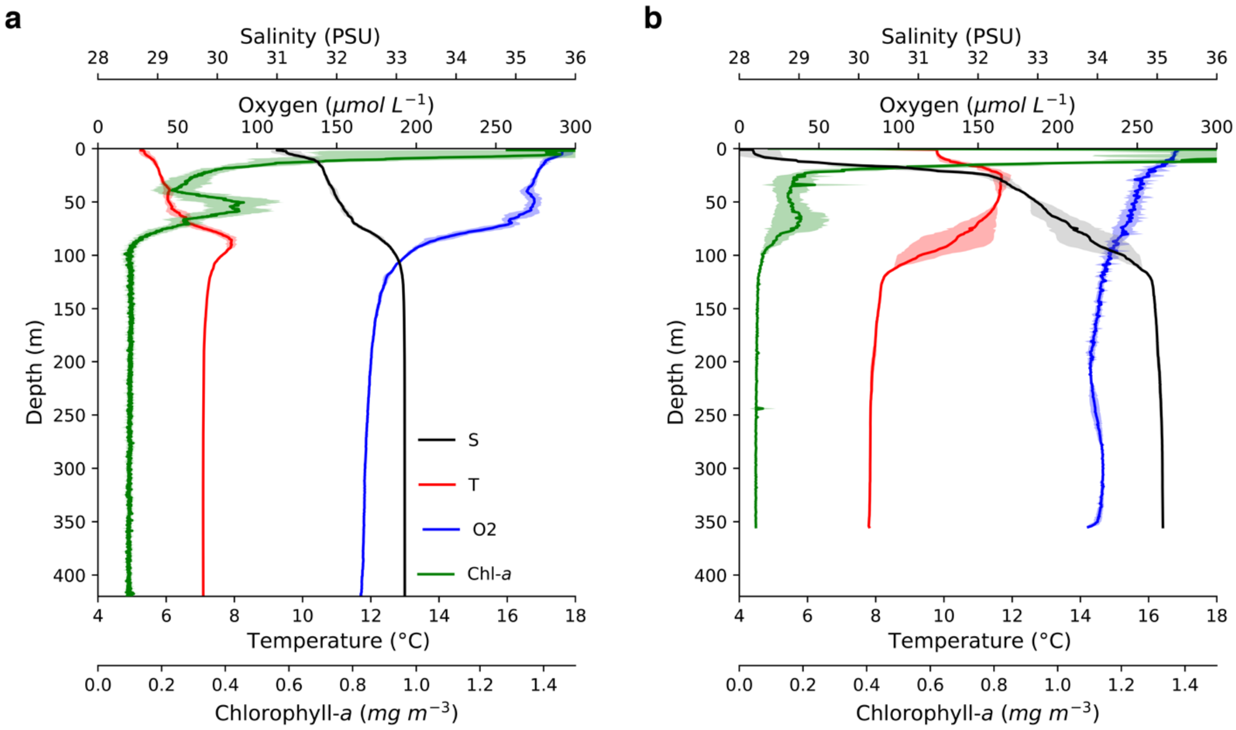

Salinity(PSU), Temperature (°C), Oxygen concentration (μmol L^{-1}) and Chlorophyll-a (Chl-a; mg m^{-3}) profiles in the (**a**) Lurefjord and (**b**) Sognefjord, Norway, during the experimental periods.

Fig. S9

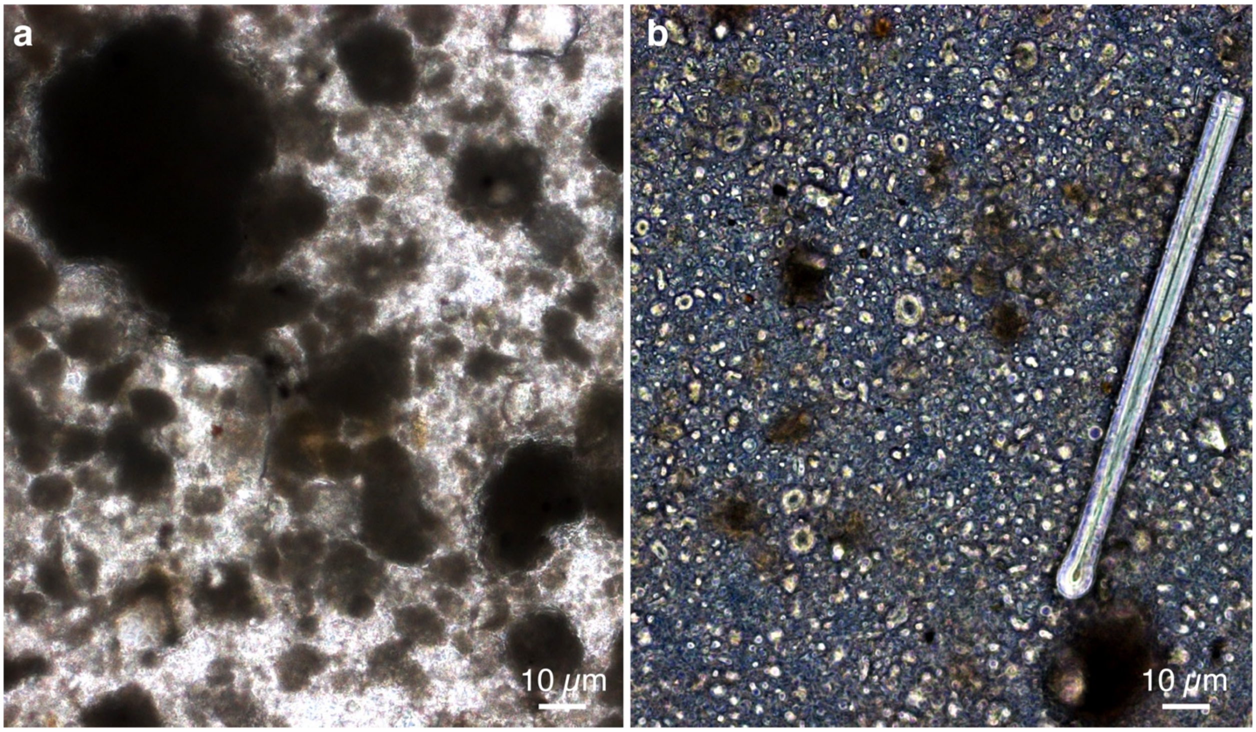

Composition of abyssal plain sediment used in the suspended sediment experiments, collected at 4427 m in the North Atlantic (47.250°N 10.105°W). **a** Organic aggregates. **b** Organic and inorganic particles, including diatom shells and sediment grains. Abyssal sediment was imaged using an inverted microscope (Zeiss Axiovert 200) with a mounted camera (Axiocam). Particle diameter was determined using ImageJ v1.52k[11].

Fig. S10.

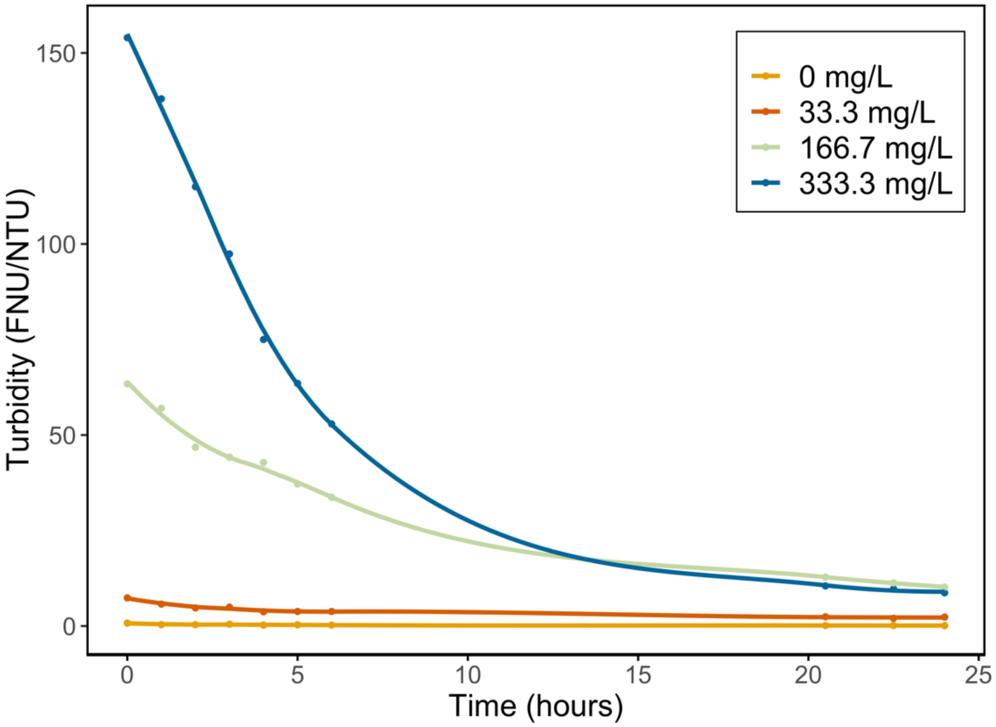

Water turbidity measured over 24 hour time period during abyssal sediment incubations (in FNU/NTU). Colors indicate sediment treatments. Turbidity was measured with a benchtop turbidity meter (WTW Turb® 340 IR). Source data are provided in the Source Data file.

Table S1. Sampling information *Periphylla periphylla* collected from Lurefjord and Sognefjord.

ID	Date caught	Fjord	Experiment	Temp. (℃)	Sediment concetration (mg/L)	Coronal diameter (cm)	Dome height (cm)	Wet Weight (g)
003	11/03/2021	Lurefjord	Temperature	7.5	NA	3.84	4.98	20.71
004	11/03/2021	Lurefjord	Temperature	7.5	NA	3.69	3.35	18.42
054	13/03/2021	Lurefjord	Sediment	7.5	166.7	2.58	3.28	6.60
055	13/03/2021	Lurefjord	Sediment	7.5	166.7	4.44	4.08	31.34
056	13/03/2021	Lurefjord	Sediment	7.5	166.7	3.67	4.04	18.23
057	13/03/2021	Lurefjord	Sediment	7.5	333.3	2.60	2.91	6.77
058	13/03/2021	Lurefjord	Sediment	7.5	333.3	2.56	2.96	6.48
059	13/03/2021	Lurefjord	Sediment	7.5	333.3	4.52	5.02	33.02
060	13/03/2021	Lurefjord	Sediment	7.5	0.0	3.05	2.85	10.74
061	13/03/2021	Lurefjord	Sediment	7.5	0.0	3.54	4.04	16.45
062	13/03/2021	Lurefjord	Sediment	7.5	0.0	5.14	6.51	47.91
063	13/03/2021	Lurefjord	Sediment	7.5	16.7	2.03	2.06	3.32
064	13/03/2021	Lurefjord	Sediment	7.5	16.7	2.45	2.64	5.73
065	13/03/2021	Lurefjord	Sediment	7.5	16.7	2.75	2.07	7.95
066	13/03/2021	Lurefjord	Sediment	7.5	16.7	7.31	7.31	131.40
067	14/03/2021	Lurefjord	Temperature	11.5	NA	2.46	2.08	5.77
068	14/03/2021	Lurefjord	Temperature	11.5	NA	2.65	2.64	7.15
069	14/03/2021	Lurefjord	Temperature	11.5	NA	4.26	4.58	27.90
070	14/03/2021	Lurefjord	Temperature	7.5	NA	3.33	3.72	13.73
071	14/03/2021	Lurefjord	Temperature	7.5	NA	3.05	3.43	10.70
072	14/03/2021	Lurefjord	Temperature	7.5	NA	2.44	2.45	5.62
073	14/03/2021	Lurefjord	Temperature	7.5	NA	2.35	1.68	5.05
074	14/03/2021	Lurefjord	Temperature	7.5	NA	2.33	2.30	4.92
075	15/03/2021	Lurefjord	Temperature	9.5	NA	3.17	2.97	11.98
076	15/03/2021	Lurefjord	Temperature	9.5	NA	2.43	1.82	5.59
077	15/03/2021	Lurefjord	Temperature	9.5	NA	3.29	2.79	13.27
078	15/03/2021	Lurefjord	Temperature	9.5	NA	2.16	1.87	3.97
079	15/03/2021	Lurefjord	Sediment	7.5	33.3	10.54	8.23	376.15
080	15/03/2021	Lurefjord	Sediment	7.5	166.7	10.18	6.68	340.17
081	15/03/2021	Lurefjord	Sediment	7.5	333.3	4.42	3.08	31.01
082	15/03/2021	Lurefjord	Sediment	7.5	333.3	3.49	3.50	15.70
083	15/03/2021	Lurefjord	Sediment	7.5	333.3	2.22	1.88	4.29
084	15/03/2021	Lurefjord	Sediment	7.5	16.7	4.92	3.48	42.09
085	15/03/2021	Lurefjord	Sediment	7.5	16.7	2.95	3.00	9.71
086	15/03/2021	Lurefjord	Sediment	7.5	16.7	2.29	1.56	4.72

087	15/03/2021	Lurefjord	Sediment	7.5	0.0	3.23	3.85	12.66
088	15/03/2021	Lurefjord	Sediment	7.5	0.0	3.40	3.47	14.59
089	15/03/2021	Lurefjord	Sediment	7.5	0.0	2.29	2.20	4.71
090	15/11/2021	Sognefjord	Temperature	9.5	NA	3.08	2.99	11.01
091	15/11/2021	Sognefjord	Temperature	9.5	NA	1.95	0.99	2.97
092	15/11/2021	Sognefjord	Temperature	9.5	NA	1.42	0.83	1.20
093	15/11/2021	Sognefjord	Temperature	9.5	NA	2.11	1.51	3.70
094	15/11/2021	Sognefjord	Temperature	9.5	NA	1.27	0.78	0.87
095	15/11/2021	Sognefjord	Temperature	9.5	NA	0.95	0.45	0.38
096	15/11/2021	Sognefjord	Temperature	9.5	NA	1.06	0.58	0.51
097	15/11/2021	Sognefjord	Sediment	7.5	166.7	7.08	7.08	120.18
098	19/11/2021	Sognefjord	Sediment	7.5	16.7	3.90	3.87	21.61
099	19/11/2021	Sognefjord	Sediment	7.5	0.0	5.33	5.91	53.14
100	19/11/2021	Sognefjord	Sediment	7.5	0.0	1.12	0.46	0.60
101	19/11/2021	Sognefjord	Sediment	7.5	333.3	6.40	5.32	89.79
102	19/11/2021	Sognefjord	Sediment	7.5	33.3	2.03	1.90	3.32
103	19/11/2021	Sognefjord	Sediment	7.5	33.3	1.54	1.22	1.52
104	19/11/2021	Sognefjord	Sediment	7.5	33.3	1.25	0.57	0.83
105	19/11/2021	Sognefjord	Sediment	7.5	33.3	1.27	0.64	0.86
106	19/11/2021	Sognefjord	Sediment	7.5	166.7	0.92	0.44	0.34
107	19/11/2021	Sognefjord	Sediment	7.5	166.7	1.03	0.49	0.47
109	19/11/2021	Sognefjord	Sediment	7.5	166.7	1.12	0.59	0.60
110	19/11/2021	Sognefjord	Sediment	7.5	166.7	0.83	0.35	0.26
111	19/11/2021	Sognefjord	Sediment	7.5	166.7	1.12	0.49	0.60
112	20/11/2021	Sognefjord	Sediment	7.5	33.3	7.94	6.24	166.57
113	20/11/2021	Sognefjord	Sediment	7.5	333.3	2.51	1.90	6.13
114	20/11/2021	Sognefjord	Sediment	7.5	333.3	1.19	0.79	0.72
115	20/11/2021	Sognefjord	Sediment	7.5	333.3	1.34	0.96	1.01
116	20/11/2021	Sognefjord	Sediment	7.5	333.3	1.35	0.89	1.03

Table S2. *Periphylla periphylla de novo* transcriptome statistics for the Trinity assembly, in addition to the trimmed Trinity transcriptome, filtered by the presence of open-reading frames identified by Transdecoder[12] and removal of contaminants.

	Trinity	Trinity trimmed
Read alignment rate (%)	96.62	34.55
Total transcripts (counts)	6,477,784	839,298
GC content (%)	41.03	44.76
N50 (base pairs)	681	495
BUSCO: complete (%)	98.8	97.8
singletons (%)	11.9	13.5
duplicates (%)	86.9	84.3
fragmented (%)	0.9	1.3
missing (%)	0.3	0.9

Table S3. KEGG Orthology (KO) pathways for significantly differently expressed genes in *Periphylla periphylla*, showing number of genes associated with each KO category. Columns indicate temperature (temp.) and sediment (sediment) treatments. Colors indicate overexpressed (orange: T1, P2, P3) and underexpressed (blue: T2, P6) genes (i.e. from Fig. S6).

KEGG BRITE genes and protein families	Temp. (°C)		Sediment (mg L^{-1})		
	T1	T2	P2	P3	P6
**Metabolism**					
Global and overview maps					
01100 Metabolic pathways	1	3	3	3	1
01110 Biosynthesis of secondary metabolites	1	1		1	
01120 Microbial metabolism in diverse environments		1			
01200 Carbon metabolism		1			
01210 2-Oxocarboxyl acid metabolism		1			
01230 Biosynthesis of amino acids		1			
01232 Nucleotide metabolism	1			1	1
01240 Biosynthesis of cofactors				1	
Lipid metabolism					
00140 Steroid hormone biosynthesis			2		
Amino acid metabolism					
00310 Lysine degradation			1		
Energy metabolism					
00190 Oxidative phosphorylation		2		1	
00720 Carbon fixation pathways in prokaryotes		1			
Carbohydrate metabolism					
00020 Citrate cycle (TCA cycle)		1			
Nucleotide metabolism					
00230 Purine metabolism	1			2	
00240 Pyrimidine metabolism				1	
Metabolism of other amino acids					
00480 Glutathione metabolism		1			
Xenobiotics biodegradation and metabolism					
00983 Drug metabolism – other enzymes				1	
**Genetic information processing**					
Translation					

03008 Ribosome biogenesis in eukaryotes	1	2			
03010 Ribosome				4	
03013 Nucleocytoplasmic transport			1		
Transcription					
03040 Spliceosome		1			
Folding, sorting and degradation					
04120 Ubiquitin mediated proteolysis			1	1	
**Environmental information processing**					
Signal transduction					
04010 MAPK signaling pathway				1	
04014 Ras signaling pathway	1	1			
04015 Rap1 signaling pathway	1				
04016 MAPK signaling pathway - plant	1			1	
04020 Calcium signaling pathway	1			1	1
04022 cGMP-PKG signaling pathway	1				
04024 cAMP signaling pathway	1				
04064 NF-kappa B signaling pathway			1		
04066 HIF-1 signaling pathway				1	
04070 Phosphatidylinositol signaling pathway	1				
04152 AMPK signaling pathway		1			
04150 mTOR signaling pathway		1			
04310 Wnt signaling pathway	1				
04330 Notch signaling pathway	2				
04371 Apelin signaling pathway	1				
**Cellular processes**					
Transport and catabolism					
04137 Mitophagy - animal				1	
04138 Autophagy – yeast		1			
04142 Lysosome		1		1	
04144 Endocytosis		1			
04145 Phagosome		1		1	
04146 Peroxisome		1		1	
Cell growth and death					
04114 Oocyte meiosis	1	1			
04216 Ferroptosis		1		1	
04217 Necroptosis		1			

04218 Cellular senescence	1				
Cellular community - eukaryotes					
04510 Focal adhesion				1	
04530 Tight junction		1			
Cell motility					
04810 Regulation of actin cytoskeleton		1		1	
**Organismal systems**					
Immune system					
04610 Complement and coagulation cascades				1	
04625 C-type lectin receptor signaling pathway	1				
04666 Fc gamma R-mediated phagocytosis		1			
Endocrine system					
04910 Insulin signaling pathway	1				
04912 GnRH signaling pathway	1				
04914 Progesterone-mediated oocyte maturation		1			
04915 Estrogen signaling pathway	1				
04916 Melanogenesis	1				
04921 Oxytocin signaling pathway	1	1			
04922 Glucagon signaling pathway	1				
04924 Renin secretion	1			1	1
04925 Aldosterone synthesis and secretion	1				
Circulatory system					
04261 Adrenergic signaling in cardiomyocytes	1				
04271 Vascular smooth muscle contraction	1				
Digestive system					
04970 Salivary secretion	1				
04971 Gastric acid secretion	1				
04972 Pancreatic secretion		2		2	
04974 Protein digestion and absorption		2		2	
04979 Cholesterol metabolism				1	
04978 Mineral absorption		1		1	
Nervous system					
04744 Dopaminergic synapse	1				
04720 Long-term potentiation	1	1			
04721 Synaptic vesicle cycle		1			
04722 Neurotrophin signaling pathway	3	1			

Sensory system					
04740 Olfactory transduction	1			1	1
04742 Taste transduction				1	1
04744 Phototransduction	1				
04745 Phototransduction – fly	1				
04750 Inflammatory mediator regulation of TRP channels	1				
Development and regeneration					
04713 Axon regeneration	1				
Environmental adaptation					
04713 Circadian entrainment	1				
04714 Thermogenesis		2		1	
04626 Plant-pathogen interaction	1				

Table S4. Two-sided PERMANOVA results of microbial community structure in *Periphylla periphylla* in response to temperature treatments, testing sample type (jellyfish/water), treatment and sampling date.

Sample*Treatment*Date	Df	Sum of Sqs	R_2	F	P-value
Sample type	1	0.81041	0.42987	23.1985	0.001*
Date	1	0.35684	0.18928	10.2148	0.001*
Treatment	1	0.01932	0.01025	0.5532	0.701
Residuals	20	0.69867	0.37060		
Total	23	1.88525	1.00000		
Treatment*Date	Df	Sum of Sqs	R_2	F	P-value
Date	1	0.36017	0.46726	8.2792	0.004**
Treatment	1	0.01912	0.02480	0.4394	0.676
Residual	9	0.39153	0.50794		
Total	11	0.77082	1.00000		
Samples from 14/03/21					
Treatment	1	0.02828	0.16083	1.1499	0.306
Residual	6	0.14753	0.83917		
Total	7	0.1758	1.00000		

Table S5. Two-sided PERMANOVA results of microbial community structure in *Periphylla periphylla* in response to sediment treatments, testing sample type (jellyfish/water/sediment), treatment and sampling date.

Sample*Treatment*Date	Df	Sum of Sqs	R_2	F	P-value
Sample type	2	1.6235	0.4514	20.1052	0.001***
Treatment	4	0.3662	0.10182	2.675	0.010**
Date	1	0.0828	0.02302	2.0507	0.088
Treatment* Date	3	0.4743	0.13187	3.91166	0.001***
Residual	26	1.0498	0.29188		
Total	36	3.5966	1.00000		
**Samples from 13/03/21**	**Df**	**Sum of Sqs**	**R_2**	**F**	**P-value**
Treatment	3	0.53563	0.61165	4.2	0.018**
Residual	8	0.34008	0.38835		
Total	11	0.87571	1.00000		
**Samples from 15/03/21**	**Df**	**Sum of Sqs**	**R_2**	**F**	**P-value**
Date	1	0.10522	0.26454	0.8992	0.535
Residual	5	0.29251	0.73546		
Total	8	0.39773	1.00000		

Table S6. Amplicon sequence variants (ASVs) uniquely shared between abyssal plain sediment samples and *Periphylla periphylla* exposed to sediment treatments, excluding control and seawater, in addition their relative abundances.

ASV id	Phylum	Genus	Percentage of total abundance				
			0 mg L^{-1}	17 mg L^{-1}	167 mg L^{-1}	333 mg L^{-1}	Sediment
0b5218e8f5dea4922229e4ca6daee5ef	Actinobacteriota	*Eggerthella*	0	0	0	0.056	0.016
981d0986557decf3c0f693f1eaefda77	Actinobacteriota	*Actinomyces*	0	0.959	0.012	0	0.412
5396df456145f53ab601b012847a8e5b	Bacteroidota	*uncultured*	0	0	0	0.259	0.036
6ddedb25b82b96e2e83f6b2bbdd8735d	Bacteroidota	*uncultured*	0	0	0	0.155	0.008
77da3efbeb6b1c9920e6122f6eead5d4	Bacteroidota	*Rikenellaceae RC9*	0	0.027	0	0.086	0.052
545e759aff088e5a28abeb943e9a3d77	Bacteroidota	*Barnesiella*	0	0.085	0	0.068	0.287
6366c46b518798181f5bca1a030b45af	Bacteroidota	*Prevotella*	0	0	0	0.056	0.016
06fbdc258737415e66246593a4e01edd	Bacteroidota	*Bacteroides*	0	0.373	0	0	1.247
56097dfd785b8777a9427e5103b81799	Euryarchaeota	*Methanobrevibacter*	0	0.039	0	0.016	0.057
e90a35b3a105f08aafef5196eb6b6ce1	Firmicutes	*Streptococcus*	0	0	0.222	1.394	0.920
06ee1193d8f3cfdfd22fdc6f07ec8628	Firmicutes	*Eubacterium*	0	0.346	0.061	1.067	0.674
2e2652ee188fee3bdca47f5c62da8d25	Firmicutes	*Ruminococcus*	0	0	0.070	0.738	0.295
68ffdac73d1ffb517dbd71df6027f0cf	Firmicutes	*Ruminococcus*	0	0	0	0.466	0.602
797196a39daf8d8f3e4e7f3ee9ff3901	Firmicutes	*uncultured*	0	0	0.052	0.290	0.198
533deb9c53f8610568fbbc2691698c60	Firmicutes	*Agathobacter*	0	0.016	0	0.108	0.598
b1b5490270b8016768e1d8ab269608e1	Firmicutes	*uncultured*	0	0	0	0.095	1.050
0e28d6a29a9f1556d654e8b54321c65b	Firmicutes	*uncultured*	0	0	0	0.032	0.008
65b602ea9b8c972fd15b69bc93ae102c	Firmicutes	*Ruminococcus*	0	0	0	0.020	0.028
a71358e982ec0dbb989b6860fc24823d	Firmicutes	*Blautia*	0	0.687	0	0	0.561
adf7074951fa2b8b04d712fafed0330e	Firmicutes	*Uncultured*	0	0.151	0.043	0	0.363
c213209f1e55f18e4dc798e3d8eec50b	Firmicutes	*Blautia*	0	0.202	0	0	0.081
cd17e3c506cf6412b4ea9dd9d0615f82	Fusobacteriota	*Fusobacterium*	0	0	0	0.329	0.182
810d050d7e5623ecc982acf48d17fc2c	Proteobacteria	*Sutterella*	0	0	0	0.178	0.170
a73f1da0de95daaeea1ab5c2d4949764	Proteobacteria	*Tepidimonas*	0	0	0.015	0	0.109

Table S7. Reported respiration rates for *Periphylla periphylla*, with converted units for comparison (asterisks indicate published values).

Species	mg O_2 g WW^{-1} h^{-1}	mg O_2 g DW^{-1} h^{-1}	µmol O_2 g WW^{-1} h^{-1}	µl O_2 mg DW^{-1} h^{-1}	Reference
Periphylla periphylla	0.003 to 0.037*	0.052 to 0.383			This study
Periphylla periphylla		0.143 to 1.0		0.1 to 0.7*	Youngbluth & Båmstedt[13]
Periphylla periphylla	0.003 ± 0.001		0.094 ± 0.017 SE*		Thuesen & Childress[14]
Atolla vanhoeffeni	0.006		0.201		Thuesen & Childress[14]
Atolla wyvillei	0.043 ± 0.014		0.134 ± 0.044 SE*		Thuesen & Childress[14]
Nausithoë rubra	0.007 ± 0.002		0.219 ± 0.048 SE*		Thuesen & Childress[14]
Paraphyllina ransoni	0.011 ± 0.003		0.333 ± 0.104 SE*		Thuesen & Childress[14]

Table S8. Reported ammonium excretion rates for *Periphylla periphylla* compared to coastal jellyfish.

Species	nmol NH_4 gWW^{-1} h^{-1}	Temperature	Reference
Periphylla periphylla	Average 28	7.5 and 9.5°C	This study
Periphylla periphylla	Average 184.7	11.5°C	This study
Aurelia aurita	Average ~19	20°C	Shimauchi & Uye[15]
Aurelia aurita[16], *Chrysaora quinquecirrha*[17,18], *Catostylus*[19], *Pelagia noctiluca*[20]	2 to 111	various	Reviewed by Pitt *et al*[16]
Aurelia aurita, *Chrysaora hysoscela*, *Chrysaora fulgida, Chrysaora pacifica*	28 to 86	16°C	Hubot *et al.*[21]

Supplementary references

1 Bode, M., Schukat, A., Hagen, W. & Auel, H. Predicting metabolic rates of calanoid copepods. *Journal of Experimental Marine Biology and Ecology* **444**, 1-7, doi:https://doi.org/10.1016/j.jembe.2013.03.003 (2013).

2 Packard, T. The measurement of respiratory electron-transport activity tn marine phytoplankton. *Journal of Marine Research* **29**, 235-244 (1971).

3 Owens, T. G. & King, F. D. The measurement of respiratory electron-transport-system activity in marine zooplankton. *Marine Biology* **30**, 27-36, doi:10.1007/BF00393750 (1975).

4 Aljbour, S. M., Zimmer, M., Al-Horani, F. A. & Kunzmann, A. Metabolic and oxidative stress responses of the jellyfish *Cassiopea* sp. to changes in seawater temperature. *Journal of Sea Research* **145**, 1-7, doi:https://doi.org/10.1016/j.seares.2018.12.002 (2019).

5 Packard, T., Devol, A. & King, F. D. The effect of temperature on the respiratory electron transport system in marine plankton. *Deep Sea Research and Oceanographic Abstracts* **22**, 237-249 (1975).

6 Kozich, J. J., Westcott, S. L., Baxter, N. T., Highlander, S. K. & Schloss, P. D. Development of a Dual-Index Sequencing Strategy and Curation Pipeline for Analyzing Amplicon Sequence Data on the MiSeq Illumina Sequencing Platform. *Applied and Environmental Microbiology* **79**, 5112-5120, doi:doi:10.1128/AEM.01043-13 (2013).

7 Muyzer, G., Waal, E. C. d. & Uitterlinden, A. G. Profiling of complex microbial populations by denaturing gradient gel electrophoresis analysis of polymerase chain reaction-amplified genes coding for 16S rRNA. *Applied and Environmental Microbiology* **59**, 695-700, doi:doi:10.1128/aem.59.3.695-700.1993 (1993).

8 Caporaso, J. G. *et al.* Global patterns of 16S rRNA diversity at a depth of millions of sequences per sample. *Proceedings of the National Academy of Sciences* **108**, 4516-4522, doi:doi:10.1073/pnas.1000080107 (2011).

9 R: A language and environment for statistical computing (R Foundation for Statistical Computing, Vienna, Austria, 2022).

10 Love, M. I., Huber, W. & Anders, S. Moderated estimation of fold change and dispersion for RNA-seq data with DESeq2. *Genome Biology* **15**, 550, doi:10.1186/s13059-014-0550-8 (2014).

11 Schneider, C. A., Rasband, W. S. & Eliceiri, K. W. NIH Image to ImageJ: 25 years of image analysis. *Nature Methods* **9**, 671-675, doi:10.1038/nmeth.2089 (2012).

12 Haas, B. J. *et al.* De novo transcript sequence reconstruction from RNA-seq using the Trinity platform for reference generation and analysis. *Nature Protocols* **8**, 1494-1512, doi:10.1038/nprot.2013.084 (2013).

13 Youngbluth, M. J. & Båmstedt, U. in *Jellyfish Blooms: Ecological and Societal Importance.* (eds J. E. Purcell, W. M. Graham, & H. J. Dumont) 321-333 (Springer Netherlands).

14 Thuesen, E. V. & Childress, J. J. Oxygen Consumption Rates and Metabolic Enzyme Activities of Oceanic California Medusae in Relation to Body Size and Habitat Depth. *The Biological Bulletin* **187**, 84-98, doi:10.2307/1542168 (1994).

15 Shimauchi, H. & Uye, S.-I. Excretion and respiration rates of the scyphomedusa *Aurelia aurita* from the Inland Sea of Japan. *Journal of Oceanography* **63**, 27-34, doi:10.1007/s10872-007-0003-z (2007).

16 Pitt, K. A. *et al.* Jellyfish Body Plans Provide Allometric Advantages beyond Low Carbon Content. *PLOS ONE* **8**, e72683, doi:10.1371/journal.pone.0072683 (2013).

17 Nemazie, D. A., Purcell, J. E. & Glibert, P. M. Ammonium excretion by gelationous zooplankton and their contribution to the ammonium requirements of microplankton in Chesapeake Bay. *Marine Biology* **116**, 451-458, doi:10.1007/BF00350062 (1993).

18 Condon, R. H., Steinberg, D. K. & Bronk, D. A. Production of dissolved organic matter and inorganic nutrients by gelatinous zooplankton in the York River estuary, Chesapeake Bay. *Journal of Plankton Research* **32**, 153-170, doi:10.1093/plankt/fbp109 (2009).

19 Pitt, K. A., Koop, K. & Rissik, D. Contrasting contributions to inorganic nutrient recycling by the co-occurring jellyfishes, *Catostylus mosaicus* and *Phyllorhiza punctata* (Scyphozoa, Rhizostomeae). *Journal of Experimental Marine Biology and Ecology* **315**, 71-86, doi:https://doi.org/10.1016/j.jembe.2004.09.007 (2005).

20 Morand, P., Carré, C. & Biggs, D. C. Feeding and metabolism of the jellyfish *Pelagia noctiluca* (Scyphomedusae, Semaeostomeae). *Journal of Plankton Research* **9**, 651-665 (1987).

21 Hubot, N. D. *et al.* Evidence of nitrification associated with globally distributed pelagic jellyfish. *Limnology and Oceanography* **n/a**, doi:https://doi.org/10.1002/lno.11736 (2021).
