# Supplementary material for: Experimental mining plumes and ocean warming trigger stress in a deep pelagic jellyfish
Source: Nat Commun. 2023 Nov 21;14:7352. doi: 10.1038/s41467-023-43023-6 (PMC10663454; doi:10.1038/s41467-023-43023-6)
Supplement: Supplementary file 3 — Reporting Summary [file 41467_2023_43023_MOESM3_ESM.pdf]

nature portfolio

Corresponding author(s): Vanessa I. Stenvers

Last updated by author(s): Oct 16, 2023

Reporting Summary

Nature Portfolio wishes to improve the reproducibility of the work that we publish. This form provides structure for consistency and transparency in reporting. For further information on Nature Portfolio policies, see our Editorial Policies and the Editorial Policy Checklist.

Statistics

For all statistical analyses, confirm that the following items are present in the figure legend, table legend, main text, or Methods section.

n/a	Confirmed	
☐	☒	The exact sample size (*n*) for each experimental group/condition, given as a discrete number and unit of measurement
☐	☒	A statement on whether measurements were taken from distinct samples or whether the same sample was measured repeatedly
☐	☒	The statistical test(s) used AND whether they are one- or two-sided<br>*Only common tests should be described solely by name; describe more complex techniques in the Methods section.*
☐	☒	A description of all covariates tested
☐	☒	A description of any assumptions or corrections, such as tests of normality and adjustment for multiple comparisons
☐	☒	A full description of the statistical parameters including central tendency (e.g. means) or other basic estimates (e.g. regression coefficient) AND variation (e.g. standard deviation) or associated estimates of uncertainty (e.g. confidence intervals)
☐	☒	For null hypothesis testing, the test statistic (e.g. *F*, *t*, *r*) with confidence intervals, effect sizes, degrees of freedom and *P* value noted<br>*Give P values as exact values whenever suitable.*
☒	☐	For Bayesian analysis, information on the choice of priors and Markov chain Monte Carlo settings
☒	☐	For hierarchical and complex designs, identification of the appropriate level for tests and full reporting of outcomes
☒	☐	Estimates of effect sizes (e.g. Cohen's *d*, Pearson's *r*), indicating how they were calculated

Our web collection on statistics for biologists contains articles on many of the points above.

Software and code

Policy information about availability of computer code

Data collection	The data in this manuscript was newly generated and no software was used to collect data.
Data analysis	All data was analyzed using open source software. Data from respiration, ammonium excretion and health scores were analysed in R v4.2.1. Measurements of jellyfish dimensions were taken in ImageJ v1.52k. Transcriptomes were analysed with SOAPnuke v3, Trinity v2.8.5, Transdecoder v5.50, Trinotate v3.2.1, Bowtie2 v2.5.0, Salmon v0.14.1, BUSCO v5.4.4, OrthoDB v09, R v4.2.1, DESeq2 v1.38.2, KEGG mapper reconstruct tool v5.0, and eggNOG v2.1.9. Microbial community composition was analysed using R v3.5.1, R v4.2.1, QIIME2 v2018.11, and QIIME2 v2019.10.

For manuscripts utilizing custom algorithms or software that are central to the research but not yet described in published literature, software must be made available to editors and reviewers. We strongly encourage code deposition in a community repository (e.g. GitHub). See the Nature Portfolio guidelines for submitting code & software for further information.

Data

Policy information about availability of data

All manuscripts must include a data availability statement. This statement should provide the following information, where applicable:

- Accession codes, unique identifiers, or web links for publicly available datasets
- A description of any restrictions on data availability
- For clinical datasets or third party data, please ensure that the statement adheres to our policy

Data for P. periphylla's physiological measurements can be found on the PANGAEA repository (https://doi.pangaea.de/10.1594/PANGAEA.957367), in addition to

the microbial community composition (https://doi.pangaea.de/10.1594/PANGAEA.957395) and the normalized expression counts for differentially expressed transcripts (i.e. including lfc values of significantly DE transcripts and annotations; https://doi.pangaea.de/10.1594/PANGAEA.962217). Transcriptome and microbiome sequences are hosted on NCBI's SRA database under BioProject PRJNA971902 with accession numbers SAMN35056874-902 (https://www.ncbi.nlm.nih.gov/bioproject/PRJNA971902) and BioProject PRJNA971258 with accession numbers SAMN35028032-079 (https://www.ncbi.nlm.nih.gov/bioproject/PRJNA971258), respectively. The assembled transcriptome is available on Figshare (https://doi.org/10.6084/m9.figshare.24114369.v1), while the BUSCO metazoan OrthoDB v09 references database is available on https://busco-data.ezlab.org/v5/data/lineages.

Research involving human participants, their data, or biological material

Policy information about studies with human participants or human data. See also policy information about sex, gender (identity/presentation), and sexual orientation and race, ethnicity and racism.

Reporting on sex and gender	N/A
Reporting on race, ethnicity, or other socially relevant groupings	N/A
Population characteristics	N/A
Recruitment	N/A
Ethics oversight	N/A

Note that full information on the approval of the study protocol must also be provided in the manuscript.

Field-specific reporting

Please select the one below that is the best fit for your research. If you are not sure, read the appropriate sections before making your selection.

☐ Life sciences ☐ Behavioural & social sciences ☒ Ecological, evolutionary & environmental sciences

For a reference copy of the document with all sections, see nature.com/documents/nr-reporting-summary-flat.pdf

Ecological, evolutionary & environmental sciences study design

All studies must disclose on these points even when the disclosure is negative.

Study description	In this study, we investigate the effects of global warming and sediment plumes from deep-sea mining on a deep pelagic jellyfish. Through a series of ex situ experiments, we measured the metabolic response (ammonium excretion and respiration), expression of stress related RNA transcripts and changes in microbial community composition. In total, 64 P. periphylla were collected, of which 21 were exposed to increasing temperatures and 43 to sediment plumes. Three temperature treatments were tested, including 7.5 (i.e. which was the in situ temperature, n=7), 9.5 (n=11), and 11.5 (n=3) degrees Celsius. Sediment plume treatments covered five concentrations, including 0 (n=8), 17 (n= 8), 33 (n=6), 167 (n=10) and 333 (n=11) mg/L abyssal sediment.
Research sample	Our research samples comprised the pelagic helmet jellyfish Periphylla periphylla. This species occurs globally from the surface down to 4000 m depth, making it a representative organism to study the effects of warming and mining in the global deep ocean. Moreover, P. periphylla is known for its high abundance in several Norwegian fjords, allowing for relatively easy and gentle collection of individuals while their biology and physiology remain relevant for oceanic populations. Organism gender was not determined since this was not the focus of our study, while only including mature, fully developed individuals (i.e. showing all morphological traits of medusa; Jarms et al. 1999. Sarsia). Size of all jellyfish was recorded to standardize measurements (Supplementary Table 1), with size also acting as a proxy for age since there is currently no straightforward way to determine age in P. periphylla (Youngbluth & Båmstedt. 2001. Hydrobiologica).
Sampling strategy	Jellyfish were caught in the Lurefjord and Sognefjord between 0 and 800 m depth in the dark, after sunset, using slow vertical hauls of conical plankton nets with a non-filtering cod-end to minimize damage. Sample sizes for each experimental run were determined by the quantity and size of jellyfish caught to allow enough space for normal behavior, similar to that observed in individuals in situ, in each of the five experimental tanks or respiration chambers, in addition to being determined by our overall ship- and sampling time. As such, kreisel tanks always contained up to four small (0.83–5.33 cm coronal diameter, CD) or one large (6.40–10.54 cm CD) P. periphylla. Sample sizes for the transcriptome and microbiome analyses were therefore also determined by tank capacity, and we made sure these were always equal to or greater than n=3 per single treatment. Similar sample size have previously been reported in transcriptome and microbiome studies (e.g. Barshis et al. 2013; PNAS; Hadaidi et al. 2017. Scientific Reports; DeLeo et al. 2018. Molecular Ecology; DeLeo et al. 2021. Frontiers in Marine Science; Breusing et al. 2022. ISME) and we can support our findings hollistically, combining physiology, gene expression and analysis of microbial symbionts.
Data collection	Data was collected by V.I. Stenvers and H. Hauss. During the shipboard experiments, respiration was measured with fiber optic oxygen meters and ammonium excretion was measured fluorometrically as an end-point-measurement. Health scores of organisms were quantified using a custom health score matrix (explained and illustrated in Methods section). Samples for microbial community composition and transcriptomics were taken from frozen tissue back ashore. Electron Transfer System (ETS) activity was measured with an Iodonitrotetrazolium reduction assay. RNA was extracted by V.I.Stenvers using the Zymo Quick-RNA Miniprep Plus kit. Microbiomes were extracted by Ina Clefsen using the DNeasy Power Soil Kit (Qiagen).

Timing and spatial scale	Jellyfish were collected in Norway, in the Lurefjord and Sognefjord, between 2–19 March and 11–23 November in 2021, respectively. Sampling dates were chosen based on ship availability and to align with colder months and shorter daylight hours, when jellyfish migrate from deeper depths to the surface at night that allows for gentle capture of specimens. Sampling sites were approximately 41 km apart.
Data exclusions	For the transcriptome and microbiome analyses in response to sediment plumes, data from the 33 mg/L sediment treatment were excluded as we could only generate data for a single replicate that prevented this data from being statistically reliable.
Reproducibility	Reproducibility was verified by repeating experiments within each field campaign, and across campaigns in March and November 2021. As such, experiments for the temperature treatments were repeated twice during the HE570 and AL658 cruises, while the sediment treatments were repeated twice during the HE570 and AL568 cruises each (for a total of four times). Detailed information on the timing and repetition of experimental runs is available on PANGAEA in the metadataset (https://doi.pangaea.de/10.1594/PANGAEA.957367).
Randomization	Organisms were assigned to experimental groups based on size, comprising up to four small (<5.33 cm in coronal diamter) or one large jellyfish (>6.40 cm in coronal diameter). Experimental groups consisting of multiple small individuals were assigned randomly. Experimental tanks, containers and treatments were assigned randomly to experimental groups.
Blinding	Blinding was not relevant for our study as we used lower invertebrates, and as we continually and manually monitored temperature and/or sediment turbidity in our experimental chambers.

Did the study involve field work? ☒ Yes ☐ No

Field work, collection and transport

Field conditions	Field work was carried out in March and November 2021, with cloudy skies, light rainfall and light snow. Seawater temperatures ranged between 5 and 12°C between months and across depths.
Location	Samples were collected in the Lurefjord (60.692°N 5.155°E) and Sognefjord (61.100°N 5.585°E), where experiments were executed aboard the ship.
Access & import/export	Field sites were accessed with research vessels (RV Heincke and RV Alkor), on which frozen samples were transported to the GEOMAR institution. Sampling permits were obtained from the Norwegian Directorate of Fisheries, comprising permit numbers 20/14555 for the cruise in March (issued 21.01.2021) and 21/73103 for the November cruise (issued 21.10.2021). For export of genetic data, we obtained an e-mail waiver (10.09.2020) from the the Norwegian Environment Agency, on behalf of the Norwegian Ministry of Climate and Environment, as the Nagoya protocol was not implemented yet during the study period.
Disturbance	Disturbance was minimal as we used net sampling to collect our specimens from the water column.

Reporting for specific materials, systems and methods

We require information from authors about some types of materials, experimental systems and methods used in many studies. Here, indicate whether each material, system or method listed is relevant to your study. If you are not sure if a list item applies to your research, read the appropriate section before selecting a response.

Materials & experimental systems

n/a	Involved in the study	
☒	☐	Antibodies
☒	☐	Eukaryotic cell lines
☒	☐	Palaeontology and archaeology
☐	☒	Animals and other organisms
☒	☐	Clinical data
☒	☐	Dual use research of concern
☒	☐	Plants

Methods

n/a	Involved in the study	
☒	☐	ChIP-seq
☒	☐	Flow cytometry
☒	☐	MRI-based neuroimaging

Animals and other research organisms

Policy information about studies involving animals; ARRIVE guidelines recommended for reporting animal research, and Sex and Gender in Research

Laboratory animals	The study did not involve laboratory animals.
Wild animals	For our study, natural populations of the helmet jellyfish Periphylla periphylla were sampled. Specimens were collected with nets, after which they were acclimated in the dark before starting the experiments. At the end of our experiments, animals were killed by

	snap freezing them at -20°C (to avoid warming of the -80 freezer) and transferring them to -80°C within 5 minutes of being first taken from their experimental container. Snap freezing was done to preserve and capture cellular processes for analysis of the transcriptome and Electron Transfer System (ETS) activity. Size of all jellyfish was recorded to standardize measurements (Supplementary Table 1), with size also acting as a proxy for age since there is currently no straightforward way to determine age in P. periphylla (Youngbluth & Båmstedt. 2001. Hydrobiologica).
Reporting on sex	Information not collected.
Field-collected samples	Individuals that were undamaged after net sampling and therefore suitable for experiments were acclimatized in seawater (collected at 300m depth) at their in situ temperature (7.5°C) for 7 to 14 hours. For the temperature incubations, this meant that jellyfish were kept in 60 L tanks, while those for the sediment plume experiments were kept in 30 L kreisel tanks (Schuran Seawater Equipment) with continuous water circulation maintained by gentle aeration from air pumps. All P. periphylla were kept in the dark and were handled or observed under red light, as P. periphylla contain a red porphyrin pigment that becomes toxic to themselves under bright light. At the end of each experiment, P. periphylla were quickly photographed (Olympus TG-6 camera) for later size determination and were then snap frozen until further processing back ashore.
Ethics oversight	Ethical approval not required as we worked with lower invertebrates.

Note that full information on the approval of the study protocol must also be provided in the manuscript.
